# Supplementary material for: Does Antenatal Lactoferrin Protect Hippocampal Development in Ovine Fetuses with Growth Restriction?
Source: Cells. 2025 Dec 9;14(24):1951. doi: 10.3390/cells14241951 (PMC12731233; doi:10.3390/cells14241951)
Supplement: Supplementary file 1 [file cells-14-01951-s001.zip › cells-3960072-supplementary.pdf]

Table S1

Antibody	Antigen retrieval	Endogenous peroxidases & protein block	Primary antibody	Secondary antibody
**NeuN**	0.01M citric acid, pH6<br>(3 x 5mins)<br>Cool 20mins	3% H_2O_2 in PBS (10mins)<br>5% NGS & 1% BSA in PBS (30mins)	Mouse anti-NeuN, 1:500<br>(Merck Millipore; AB377)	Goat anti-Mouse,<br>1:200<br>(Biotynylated, Vector Labs; BA-9200-1.5)
**Iba-1**	0.01M citric acid, pH6<br>(3 x 3mins)<br>Cool 20mins	0.3% H_2O_2 in 50% MeOH (30mins)<br>10% NGS in PBS (30mins)	Rabbit anti-Iba-1, 1:1000<br>(Wako Pure Chemical Industries; 019-19741)	Goat anti-Rabbit,<br>1:200<br>(Biotynylated, Vector Labs; BA-9100-1.5)
**8-OHdG**	0.01M citric acid, pH6<br>(3 x 5mins)<br>Cool 20mins	0.3% H_2O_2 in 50% MeOH (15mins)<br>5% NGS & 3% BSA in 0.1% PBS-TX (45mins)	Mouse anti-8-OHdG, 1:200<br>(JalCA; MOG-100P)	Goat anti-Mouse,<br>1:200<br>(Biotynylated, Vector Labs; BA-9200-1.5)
**BDNF**	0.05% Tween 20 in 10mM citrate, pH6<br>(3 x 5mins)<br>Cool 15mins	0.3% H_2O_2 in 50% MeOH (15mins)<br>10mg/ml $NaBH_4$ in PBS (3 x 10mins)<br>20% NGS & 1% BSA in 0.1% PBS-TX (60mins)	Rabbit anti-BDNF, 1:500<br>(Abcam; AB108319)	Goat anti-Rabbit, 1:300 (Invitrogen, Alexa Fluor 594)
**MAP2**	0.1% TBS-TX, pH10<br>(2 x 10mins)	3% H_2O_2 in PBS (10mins)<br>10mg/ml $NaBH_4$ in PBS (2 x 15mins)<br>DAKO protein block serum (30mins)	Mouse anti-MAP2,<br>1:200<br>(Invitrogen; MA1-25044)	Goat anti-Mouse, 1:1000 (Invitrogen, Alexa Fluor A488)
